# A systematic review of wellbeing in children: a comparison of military and civilian families

**DOI:** 10.1186/s13034-018-0252-1

**Published:** 2018-11-07

**Authors:** Victoria Williamson, Sharon A. M. Stevelink, Eve Da Silva, Nicola T. Fear

**Affiliations:** 1 Kings Centre for Military Health Research, King’s College London, Weston Education Centre, 10 Cutcombe Road, London, SE5 9RJ UK; 2 Academic Department for Military Mental Health, King’s College London, Weston Education Centre, 10 Cutcombe Road, London, SE5 9RJ UK

**Keywords:** Child, Parent, Military, Systematic review, Wellbeing, Sibling

## Abstract

**Background:**

Children in military families have uniquely different childhood experiences compared to their civilian peers, including a parent in employment and a stable familial income, frequent relocations, indirect exposure to and awareness of conflict, and extended separation from parents or siblings due to deployment. However, whether children from military families have poorer wellbeing than non-military connected children is not well understood.

**Method:**

We conducted a systematic review to explore the relationship between military family membership (e.g. parent or sibling in the military) and child wellbeing compared to non-military connected controls. Searches for this review were conducted in September 2016 and then updated in February 2018.

**Results:**

Nine studies were identified, eight were cross-sectional. All studies utilised self-report measures administered in US school settings. On the whole, military connected youth were not found to have poorer wellbeing than civilian children, although those with deployed parents and older military connected children were at greater risk of some adjustment difficulties (e.g. substance use, externalising behaviour). Although only assessed in two studies, having a sibling in the military and experiencing sibling deployment was statistically significantly associated with substance use and depressive symptoms.

**Conclusions:**

This study is unique in its direct comparison of military and non-military connected youth. Our results highlight the need to examine the impact of military service in siblings and other close relatives on child wellbeing. Given the adverse impact of poor mental health on child functioning, additional research is needed ensure appropriate, evidence-based interventions are available for youth in military families.

## Background

Children in military families experience frequent separation from parents and/or siblings due to deployment or operations, regular moves and relocations, indirect exposure to and awareness of conflict and violence, and exposure to a family member who may return from combat with psychological or physical injuries [1]. However, these children also experience particular benefits, such as a parent in employment and, thus, a stable family income.

To date, studies examining the impact of military family membership on child psychological adjustment and wellbeing have yielded mixed results (e.g. [2–7, 8]). Thus, how military family membership may impact child psychological wellbeing, including externalising behaviours such as physical fighting and weapon carrying, substance use, and mental health problems, as compared to their peers in civilian families remains unclear.

Child externalising behaviours are associated not only with concurrent health problems, lower educational attainment, but also violent behaviour in adulthood (for a review, see [2]). In civilian families, externalising behaviours are more commonly observed in male children and can be associated with rejection by peers and low socio-economic status [3, 4]. In military families, some studies have observed children are more likely to exhibit externalising behaviours when the parent is deployed due to heightened anxiety regarding the deployment situation and the service member’s safety [5–7]; however, this deleterious effect of deployment on child externalising behaviours has not been consistently found [9, 10].

Another key component to child psychological wellbeing is substance use, with early consumption of alcohol and drugs associated with increased risk of dependence later in adulthood [11, 12]. Nationally representative studies in the US have found the prevalence of substance use disorders in children aged 13–18 years to be 11.4%, with substance misuse more common in males and older adolescents [13]. In military families, young people with deployed parents have been found to be more likely to consume alcohol and binge drink than their civilian peers [14, 15]. Nonetheless, beyond parental deployment, how other factors, such as age and gender, may moderate the relationship between military family membership and child substance use as compared to children in civilian families remains unclear.

Finally, the experience of other mental health problems in childhood, including depression, post-traumatic stress disorder (PTSD) and suicidality, can adversely affect wellbeing. Young people in military families may potentially be vulnerable to mental health problems due to their exposure to a range of stressors, including a parent with mental health difficulties (i.e. military-service related psychological problems, non-deployed parent coping difficulties, etc.) [16], frequent relocations, or the reintegration of the deployed parent. However, as youth in civilian families are also exposed to challenging circumstances, such as poor parental mental health [17], how the mental health of young people in military families compares to children in civilian families is not well understood.

Little research attention has been given to the impact of having a sibling in the military on child wellbeing. Previous studies have found sibling relationships to have developmental significance, with sibling relationship difficulties linked to a range of poor outcomes in children, including depression, low family functioning, aggression, substance use, and delinquency [18]. Evidence from qualitative studies highlights that sibling enlistment may be particularly challenging for children in military families, with difficulties including family role shifts on sibling enlistment, increased loneliness, and concerns that their sibling may be injured on deployment [19]. Sibling enlistment can also be distressing for the family unit as a whole, increasing familial conflict and causing significant parental distress [19]. As poor family functioning and shifts in familial roles have been found to adversely impact child wellbeing in civilian families (e.g. [20–22]), how sibling enlistment impacts wellbeing compared to children of military parents and children in civilian families is poorly understood.

Taken together, it is unclear how children in military families compare to their non-military peers in terms of wellbeing. The aim of this review was to examine the association between military family membership and child wellbeing compared to non-military controls. We also considered several moderators of child outcomes, including child age, gender, and methodological factors.

## Method

### Search strategy

Electronic literature databases were searched in September 2016 and again in February 2018 for relevant studies, including PsycInfo, EMBASE, MEDLINE, PubMed, Google Scholar, and Web of Science. Search terms included military (military OR army OR combat OR armed forces OR soldier OR navy OR air force OR marine OR veteran OR service personnel OR sailor OR airman OR military personnel OR military deployment), child (child* OR famil* OR offspring OR adolescen*) and wellbeing (resilien* OR hardiness OR wellbeing OR mental OR well-being OR health*) key words. Reference lists of relevant articles and review papers (e.g. [7]) and issues of journals (e.g. Journal of Traumatic Stress; Journal of Adolescent Health) were also examined for eligible studies.

### Eligibility

To be considered for inclusion, studies had to include: a sample of children with a parent or sibling in the military compared to a sample of children without a military connected parent/sibling; a measure of child mental health or wellbeing; and a sample of child participants below 19 years of age. Excluded studies included:

- Case studies.
- Reviews.
- Studies which only presented qualitative findings.
- Studies not written in English.
- Studies where there was no comparison provided between children from military families and non-military families.
- Conference abstracts and Ph.D. dissertations where additional information or published versions could not be found or obtained from the corresponding author.

**Fig. 1:**
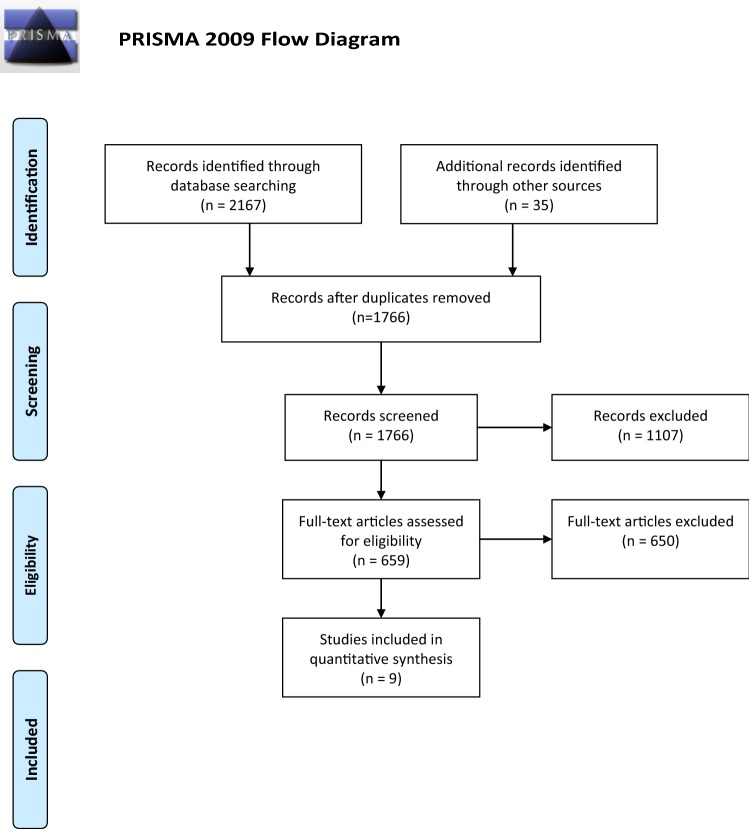
PRISMA Flow chart

We use the term ‘child’ throughout to refer to both children and adolescents under the age of 19 years. A child in a military family was defined as the legal dependent of a military serviceman/woman (of any nationality) or a child with a sibling in the AF. A Preferred Reporting Items for Systematic Reviews and Meta-Analyses (PRISMA) flow chart (Fig. 1) describes the systematic review process [23]. Nine studies met the inclusion criteria for this review.

### Data extraction

The following data was extracted from each study, if available: (a) study information (e.g. study design, location); (b) child demographic information (age, family status [e.g. military, non-military], ethnicity, sex); (c) the assessment time points and retention rates for longitudinal studies; (d) aspect of child wellbeing assessed; (e) how wellbeing was measured (i.e. questionnaire, interview); (f) child wellbeing informant (i.e. child, parent, teacher); (g) findings; (h) ethical issues; (i) and sources of bias. Two authors (VW and SAMS) independently extracted and assessed data for accuracy. Any discrepancies were discussed and resolved.

### Data synthesis

The following child wellbeing outcomes were explored in this review: the prevalence of child mental health disorders (PTSD, depression, suicidal ideation, and substance use), quality of life (perceived stress, positive affect, quality of life) and externalising behaviour (physical fighting, carrying a weapon). We separately examined outcomes for: (i) children in civilian families, (ii) children with a primary caregiver in the military, (iii) children with a caregiver in the military who was deployed to a combat zone, and (iv) children with a sibling in the military. If child outcomes were available for pre- and post-parental deployment or at commencement or cease of major hostilities [24], we used the rate of child mental health disorders/behaviour problems post-deployment and following the cease of major hostilities to allow for this data to be compared to studies that did not make this distinction. We also examined whether there were any differences in child outcomes pre/post major hostilities [24]. Odds ratios (OR) or adjusted odds ratios (AOR) and 95% confidence intervals (CI), were extracted from the studies. Where the OR were not available, unadjusted OR were calculated from the data. The reference category for all effect sizes was having a civilian parent. For all studies, effect sizes were regarded as statistically significant at p = 0.05 if the 95% CI did not include 1.

### Study quality

The methodological quality of studies was independently assessed by two authors (VW and SAMS) using a 14-item checklist [25]. Studies were scored depending on whether they met the specific criteria (‘no’ = 0, ‘yes’ = 1). Studies had to at least meet criteria for items three (“Was the participation rate of eligible persons at least 50%?”), eleven (“Were the outcome measures (dependent variables) clearly defined, valid, reliable, and implemented consistently across all study participants?”), and fourteen (“Were key potential confounding variables measured and adjusted statistically for their impact on the relationship between exposure(s) and outcome(s)?”; see [25]) to receive a quality score of ‘good.’ A study that met criteria on at least two of three items received a quality rating score of ‘fair’, while a study that met one or none of these items received a score of ‘poor.’ There was good agreement between reviewers. Any disagreements in quality rating scores were resolved following a re-examination of the data and discussion in a consensus meeting. Study quality ratings are provided in Table 1.

**Table 1:** Included studies sample characteristics, methods of assessment, and quality ratings

Study	Design	*N*	Males (%)	Child ethnicity (%)	Child age or school grade	Outcomes assessed	Quality rating
Acion et al. [28]	Cross-sectional	Civilian 57,637	49.3	85.9	6th, 8th, 11th grade	Alcohol/drug use in last 30 days	Good
		Deployed 1758					
Barnes et al. [24]	Longitudinal	Civilian 53	51.7	25.6	*M* 15.8 years (*SD* 1.1)	Stress, PTSD	Good
		Military parent 59					
		Deployed 21					
Cederbaum et al. [31]^a^	Cross-sectional	Civilian 12,385	48.1	28.3	7th, 9th, 11th grade	Suicidal ideation, positive affect, depression	Fair
		Military parent 1305					
		Military sibling 609					
Gilreath et al. [15]^a^	Cross-sectional	Civilian 12,555	47.9	28.2	7th, 9th, 11th grade	Alcohol/drug use in last 30 days	Good
		Military parent 1338					
		Military sibling 619					
Gilreath et al. [29]^a^	Cross-sectional	Civilian 283,593	49.1	23.5	9–11th grade	Suicidal ideation	Good
		Military parent 27,547					
Reed et al. [14]^b^	Cross-sectional	Civilian 8237	57.2	N/A	8th, 10th, 12th grade	Quality of life, depression, suicidal ideation	Good
		Military parent 1216					
		Deployed 557					
Reed et al. [26]^b^	Cross-sectional	Civilian 9978	56.0	60.5	8th, 10th, 12th grade	Binge drinking over last 2 weeks, drug use in last 30 days, externalising behaviour	Good
		Military parent 1210					
		Deployed 554					
Reinhardt et al. [30]	Cross-sectional	Civilian 3370	49.6	36.0	9–12th grade	Externalising behaviour	Good
		Military parent 539					
Sullivan et al. [27]^a^	Cross-sectional	Civilian 634,029	49.6	21.4	7th, 9th, 11th grade	Externalising behaviour, alcohol/drug use in last 30 days	Good
		Military parent 54,684					

*N *= total number of child participants. Child ethnicity is reported as percentage Caucasian children. Males = the percentage of male children in the study. Military parent/sibling = child reports having a primary caregiver or sibling in the armed forces. Deployed = child reports that parent/sibling has been deployed to a combat zone. *N/A* not available, *M* mean, *SD* standard deviation. Adjustment difficulties measured = type of child psychological difficulty assessed by the study and included in the analysis. Quality rating score: studies meeting criteria for items three, eleven and fourteen on the NIH [25] study quality checklist received a score of ‘good.’ A study that met criteria on two of three items received a quality rating score of ‘fair.’ A study that met one or none of these items received a score of ‘poor.’ All studies assessed child wellbeing using self-report questionnaires

^a^Data from the state-wide California Healthy Kids Survey (CHKS) was used. Cederbaum et al. [31] reported CHKS data from children recruited during 2011. Gilreath et al. [15] reported CHKS data from a sub-sample of children recruited during February–March 2011 from schools in southern California. Gilreath et al. [29] used CHKS data from children recruited between 2012 and 2013. Sullivan et al. [27] reported CHKS data collected during March–April 2013

^b^Data from the Washington State 2008 Healthy Youth Survey (HYS) was used. Reed et al. [14] reported on HYS data collected in 2008, with data regarding suicidal ideation and poor quality of life used for the present study. Reed et al. [26] reported HYS data collected in 2008 with data regarding child violent behaviour and substance use used for the present study

## Results

### Study sample

The nine studies identified were published between 2007 and 2016. Study quality ratings ranged from ‘good’ to ‘fair’. All studies were conducted in the US and recruited children via schools. Children were all in 6–12th grade (11–18 years, see Table 1). All studies collected data on child wellbeing using child self-report, often using non-validated measures [15, 26–30] or questionnaires adapted from other measures [14, 24, 31]. Six studies [14, 15, 26, 27, 29, 31] used data from large-scale public-school surveys conducted in several waves (i.e. Washington State 2008 Healthy Youth Survey (HYS), [32]; California Healthy Kids Survey (CHKS), [33]).^1^ Eight studies were cross-sectional [14, 15, 26–31] and one study was longitudinal [24]. In all but three studies, information regarding parental deployment was provided [27, 29, 30]. Only two studies reported information about sibling service in the military [15, 31].

### Military connected children and externalising behaviour

Three studies reported externalising behaviour data regarding school-based physical fighting and carrying a weapon (Table 2). Sullivan and colleagues [27] found that significantly more children with parents in the military reported having been in physical fights (AOR 1.67; 95% CI 1.62, 1.71) and carrying a weapon (AOR, 1.90; 95% CI 1.83, 1.97) than civilian children in the past 12 months. This is consistent with Reinhart et al. [30] (AOR 1.69; 95% CI 1.27, 2.25). Differences in physical fighting and carrying a weapon were largely non-significant between younger children with a civilian parent and military-connected (both deployed and non-deployed) children in Reed et al. study [26]. The only exception to this was in 8th grade males with a deployed parent who reported significantly more physical fighting compared to children with civilian parents (AOR 1.57; 95% CI 1.00, 2.47). In older children (10th/12th grade), those with deployed and military (non-deployed) parents were significantly more likely than civilian children to engage in physical fighting (see Table 2). However, significant differences between groups in terms of weapon carrying were only observed in older males with deployed parents (AOR, 2.27; 95% CI 1.48, 3.47) and females with non-deployed military parents (AOR 2.03; 95% CI 1.15, 3.59).

**Table 2:** Externalising behaviour in military and non-military connected children

Study		Physical fighting	Carrying a weapon		
Reinhardt et al. [30]^a^	AOR Overall (95% CI)	1.69* (1.27, 2.25)			
	Parent military				
	Male AOR (95% CI)	1.74* (1.15, 2.65)			
	Female AOR (95% CI)	1.65* (1.11, 2.45)			
Sullivan et al. [27]^b^	AOR Overall (95% CI)	1.67* (1.62, 1.71)	1.90* (1.83, 1.97)		

		8th grade	10th/12th grade	8th grade	10th/12th
Reed et al. [26]^c^	Military parent				
	Male AOR (95% CI)	1.27 (0.92, 1.76)	1.38* (1.02, 1.85)	1.18 (0.69, 2.00)	1.08^d^ (0.74, 1.59)
	Female AOR (95% CI)	0.96 (0.60, 1.55)	2.16* (1.15, 2.85)	1.32 (0.64, 2.75)	2.03* (1.15, 3.59)
	Deployed parent				
	Male AOR (95% CI)	1.57* (1.00, 2.47)	2.01* (1.39, 2.90)	0.86 (0.39, 1.94)	2.27* (1.48, 3.47)
	Female AOR (95% CI)	1.29 (0.65, 2.58)	1.99* (1.09, 3.65)	1.62 (0.78, 3.43)	1.64 (0.77, 3.51)

*CI* confidence interval, *AOR* adjusted odds ratio. For AOR the reference category was children of civilian parents. Male and female refers to the gender of the child

* Confidence intervals indicate a statistically significant odds or adjusted odds ratio

^a^AOR adjusted for sex, race/ethnicity, grade, location substance use, depressive symptoms, and bullying victimization

^b^AOR adjusted for sex, race/ethnicity and grade

^c^AOR adjusted for race/ethnicity, grade, maternal education, academic achievement, binge drinking, drug use and media use. Reinhardt et al. [30] assessed violent behaviour using the following item: “how many times were you in a physical fight in the last 12 months?”. Reed et al. [26] assessed violent behaviour using the following items: “during the past 12 months how many times were you in a fight on school property?” and “during the past 30 days, how many times did you carry a weapon, such as a gun, knife or club on school property?”. Sullivan et al. [27] assessed in-school violent behaviour with items including: “during the past 12 months, how many times on school property have you been in a fight?”, “during the past 12 months, how many times on school property have you carried a gun?”, and “during the past 12 months, how many times on school property have you carried any other weapon (such as a knife or club)?”

^d^Difference between military and deployed significant at p < 0.05

### Military connected children and substance use

Four studies reported child substance use, including tobacco, alcohol consumption, marijuana, and other drug use (Table 3).

**Table 3:** Substance use in military and non-military connected children

Study		Alcohol	Other drugs	Tobacco	Marijuana		
Acion et al. [28]	OR (95% CI)	1.67* (1.49, 1.87)	3.52* (2.99, 4.14)		2.19* (1.87, 2.58)		
Gilreath et al. [15]	Parent military OR (95% CI)	0.91 (0.79, 1.04)	1.28* (1.04, 1.57)	1.04 (0.85, 1.29)	0.96 (0.81, 1.13)		
	Sibling military OR (95% CI)	1.18^c^ (0.98, 1.43)	1.00 (0.72, 1.38)	1.19 (0.89, 1.58)	1.09 (0.87, 1.37)		
Sullivan et al. [27]^a^	AOR Overall (95% CI)	1.50* (1.46, 1.55)	1.73* (1.66, 1.80)	1.59* (1.53, 1.66)	1.45* (1.40, 1.50)		

		8th grade	10th/12th grade	8th grade	10th/12th grade		
Reed et al. [26]^b^	Parent military						
	Male OR (95% CI)	1.28 (0.83, 1.97)	1.65* (1.30, 2.08)	1.23 (0.82, 1.83)	1.67* (1.32, 2.11)		
	Female OR (95% CI)	1.01 (0.61, 1.64)	1.86* (1.44, 2.39)	1.64* (1.02, 2.62)	1.50* (1.15, 1.94)		
	Parent deployed						
	Male OR (95% CI)	1.87* (1.15, 3.03)	1.65* (1.15, 2.35)	1.34 (0.82, 2.19)	2.08* (1.47, 2.94)		
	Female OR (95% CI)	1.93*^d^ (1.15, 3.21)	1.86* (1.24, 2.79)	1.48 (0.79, 2.74)	1.92* (1.28, 2.85)		

*AOR* adjusted odds ratio, *OR* unadjusted odds ratios. For OR and AOR the reference category was children of civilian parents. *CI* confidence intervals

* Confidence intervals indicate a statistically significant odds or adjusted odds ratio

^a^AOR adjusted for sex, race/ethnicity and grade

^b^Alcohol consumption is a measure of self-reported binge drinking over the last 2 weeks, all other studies assessed substance use in the last 30 days

^c^Difference between parent military and sibling military significant at p < 0.05

^d^Difference between military and deployed significant at p < 0.05

Children with civilian parents were found to have lower rates of alcohol and drug consumption compared to military-connected youth as reported by Sullivan et al. [27] and Acion et al. [28]. While Reed et al. [26] found older children (10th/12th grade) in military connected families (both non-deployed and deployed parents) to report significantly greater drug and alcohol use than civilian children with no associated observations for younger children (8th grade), irrespective of gender. Although no significant differences in alcohol consumption between those with a military parent vs civilian parent were found, younger children (8th grade) with a deployed parent were statistically more likely to consume alcohol than civilian children (Male OR 1.87; 95% CI 1.15, 3.03; Female OR 1.93; 95% CI 1.15, 3.21). No statistically significant differences in alcohol and drug consumption between military (non-deployed) and deployed parental groups were observed, with the exception of significantly greater alcohol consumption in younger (8th grade) females with deployed parents (OR 1.98; 95% CI 1.01, 3.88 [data not shown in table]).

Gilreath et al. [15] found no significant differences in alcohol, marijuana and tobacco consumption between children with civilian and military parents. The only significant association found was in terms of illicit drug use (e.g. crack/cocaine, inhalants, methamphetamine, LSD, etc.) and children with a parent in the military were significantly more likely to report consumption than children with a civilian parent (OR 1.28; 95% CI 1.04, 1.57). Children with a sibling in the military were significantly more likely to consume alcohol than children with a parent in the military (OR 1.30; 95% CI 1.04, 1.64, [data not shown in table]), although those with a sibling in the military were not significantly more likely to consume alcohol compared to children with civilian parents (OR 1.18; 95% CI 0.98, 1.43; [15]). However, it should be noted that this effect is approaching significance.

### Military connected children and mental health

Three studies examined child self-report of suicidal ideation over the last 12-months [14, 29, 31], with two of these studies also reporting data on child self-reported depression [14, 31]. One study examined child PTSD [24]. Three studies examined child wellbeing more generally (perceptions of stress, [14]; positive affect, [31]; poor quality of life, [14]).

#### Suicidal ideation

Cederbaum et al. [31] did not find military connectedness (i.e. parent in the military or sibling in the military) to be significantly associated with suicidal ideation (parent AOR 1.10; 95% CI 0.88, 1.38; sibling AOR 1.21; 95% CI 0.98, 1.48). Although, it should be noted that the suicidal ideation—sibling in the military AOR is approaching significance. In Reed et al. [14] study, significantly higher rates of suicidal ideation were only found in male youth (8th/10th/12th grade) and younger females (8th grade) with a deployed parent compared to civilian children ([21]; see Table 4). One study [29] found military connectedness to be significantly associated with higher rates of child suicidal ideation (AOR 1.43; 95% CI 1.37, 1.49).

**Table 4:** Mental health in military and non-military connected youth

Study		Depression	Suicidal ideation		
Cederbaum et al. [31]^a^	Parent military AOR Overall (95% CI)	0.90 (0.81, 1.01)	1.10 (0.88, 1.38)		
	Sibling military AOR Overall (95% CI)	1.13 (0.94, 1.36)	1.21 (0.98, 1.48)		
Gilreath et al. [29]^b^	AOR (95% CI)		1.43* (1.37, 1.49)		

		8^th^ grade	10^th^/12^th^ grade	8^th^ grade	10^th^/12^th^ grade
Reed et al. [14]^c^	Parent military				
	Male AOR (95% CI)	1.00 (0.70, 1.44)	1.05 (0.80, 1.39)	1.12 (0.69, 1.82)	1.27 (0.96, 1.69)
	Female AOR (95% CI)	1.22 (0.93, 1.60)	0.91 (0.73, 1.13)	1.04^±^ (0.69, 1.55)	1.13 (0.86, 1.46)
	Parent deployed				
	Male AOR (95% CI)	1.37 (0.96, 1.95)	1.50* (1.02, 2.20)	1.75* (1.15, 2.67)	1.64* (1.13, 2.38)
	Female AOR (95% CI)	1.37 (0.95, 1.97)	1.24 (0.87, 1.76)	1.66*^d^ (1.19, 2.32)	1.08 (0.70, 1.68)

*AOR* adjusted odds ratio. The reference category for the adjusted odds ratio was children of civilian parents. *CI* confidence intervals, *PTSD* posttraumatic stress disorder, *M* mean, *SD* standard deviation

* Confidence intervals indicate a statistically significant odds or adjusted odds ratio

^a^AOR adjusted for study design

^b^AOR adjusted for grade, sex, and race/ethnicity

^c^AOR adjusted for race/ethnicity, grade, maternal education, academic achievement, binge drinking, and drug use

^d^Difference between military and deployed significant at p < 0.05

#### Depression

Military connectedness (i.e. sibling or parent in the military) was not significantly associated with depressive symptoms ([31]; see Table 4). However, an increased likelihood of depressive symptoms in youth who experienced the deployment of a family member (i.e. parent, sibling) compared to those who had not experienced familial deployment was observed (AOR 1.15, 95% CI 1.00, 1.33; [31] [data not shown in table]). Similar findings were reported by Reed et al. [14], where no significant differences in depressive symptoms were found between military and non-military connected youth, except in cases of parental deployment. Older males (10^th^/12^th^ grade) who experienced parental deployment reported significantly more depressive symptoms than civilian children (AOR 1.50; 95% CI 1.02, 2.20). This was not observed in females.

#### PTSD

Child PTSD symptoms were reported in one study [24] and, following the cease of major hostilities in May 2003, children whose parents were deployed reported significantly higher levels of PTSD symptoms (mean score on Post-traumatic Stress Disorder Checklist [PCL-C; [34]] = 28.9, *SD *= 5.51) compared to children whose parents were in the military but did not deploy (mean score = 23.1, *SD *= 0.21, *p *< 0.0001) and civilian children (mean score = 20.1, *SD *= 0.17, *p *< 0.0001). Information regarding the number of children meeting case criteria for likely PTSD pre/post major hostilities in this study was not available.

#### Quality of life

Child perceptions of stress were measured by Barnes et al. [24]. Following the cease of major hostilities in May 2003, youth with a deployed parent reported significantly more stress (mean score on Psychosocial Resources Scale [PRS; [35]] = 28.0, *SD *= 4.42) than children with non-deployed parents (PRS mean score = 23.4, *SD *= 0.78, *p *< 0.0001) and children with civilian parents (PRS mean score = 22.5, *SD *= 0.56, *p *< 0.0001). These data must be interpreted cautiously as youth with deployed parents also reported the highest rates of stress pre-major hostilities. Reed et al. ([14]; see Table 5) examined poor quality of life and, in general, military connectedness was not significantly associated with poorer quality of life. However, male children (8th/10th/12th grade) whose parents had deployed reported significantly poorer quality of life compared to male children with a (non-deployed) military or civilian parent.

**Table 5:** Quality of life in military and non-military connected youth

Study	Quality of life	Positive affect	Stress *M* (SD)
Barnes et al. [24]^a^	Civilian		22.5 (0.56)
	Parent military		23.4 (0.78)
	Parent deployed		28.0 (4.42)
Cederbaum et al. [31]^b^	Parent military AOR Overall (95% CI)	0.79 (0.67, 0.94)	
	Sibling military AOR Overall (95% CI)	0.91 (0.69, 1.21)	

		8th grade	10th/12th grade
Reed et al. [14]^c^	Parent military		
	Male AOR (95% CI)	1.28^d^ (0.91, 1.79)	1.72*^d^ (1.31, 2.26)
	Female AOR (95% CI)	0.99 (0.72, 1.36)	1.21 (0.94, 1.55)
	Parent deployed		
	Male AOR (95% CI)	2.10*^d^ (1.43, 3.10)	2.74*^d^ (1.79, 4.20)
	Female AOR (95% CI)	1.21 (0.84, 1.82)	1.13 (0.74, 1.76)

*AOR* adjusted odds ratio. The reference category for the adjusted odds ratio was children of civilian parents. *M* mean, *SD* standard deviation, *CI* confidence intervals

* Confidence intervals indicate a statistically significant odds or adjusted odds ratio

^a^Score reflects child self-reported mean score on the Psychosocial Resources Scale on May 15th and 16th 2003 at the declaration of the end of “major hostilities” of Operation Iraqi Freedom

^b^AOR adjusted for study design

^c^AOR adjusted for race/ethnicity, grade, maternal education, academic achievement, binge drinking, and drug use. Poor quality of life assessed via Youth Quality of Life Instrument Surveillance Version (e.g. “I feel alone in my life”; [36])

^d^Difference between military and deployed significant at p < 0.05

## Discussion

The aim of this review was to examine the impact of military family membership on child wellbeing compared to children from non-military families. We examined child wellbeing in relation to externalising behaviour, substance use, and mental health problems. The findings of this review show the existing literature to be heterogeneous, largely involving children recruited from US public schools, where children completed self-report questionnaires to measure wellbeing. The main finding was that, overall, there was little difference between military and non-military connected children, except those with deployed parents and older military-connected children were at greater risk of substance use and externalising behaviour. Moreover, while only assessed by two studies, having a sibling in the military and experiencing sibling deployment was associated with substance use and depressive symptoms.

### Externalising behaviour

A relationship was found between military family membership and higher rates of externalising behaviour compared to children from civilian families, including engagement in physical fights and carrying a weapon [26, 27, 30]. However, this effect appeared to be moderated by child demographic characteristics (i.e. older age, male gender) and parental deployment [26].

Externalising behaviours are an important component to consider in examining child psychological wellbeing given the relationship between externalising problems and issues later in child and adulthood (e.g. crime, low education attainment, etc.) [2]. It is possible that the elevated rates of violent behaviour found in some children from military families is due to these youths being disproportionately influenced by portrayals of the military and war in the media, which emphasises physical fighting and weapons [26]. Furthermore, US military connected children may have increased access to weapons as, compared to civilians, military personnel are more likely to have a firearm in their home [37]. These results tentatively suggest that additional support, such as violence prevention programs, for some children within military families could be beneficial.

### Substance use

Substance use in childhood represents a concern as 90% of US adults with chronic substance abuse problems report starting drinking or using drugs before the age of 18 years [38]. Three studies found higher rates of substance use in youth who had experienced the deployment of a family member [15, 26, 28] compared to those with non-deployed or civilian parents. The deployment of a family member can be a substantial stressor for some children and alcohol and drugs may be utilised as a coping strategy [39]. Lower levels of parental monitoring during adolescence are associated with higher levels of child drug and alcohol use [40, 41] and parental deployment may also reduce the availability of the non-deployed parent due to increased household responsibilities [42]. As older children in military families were more likely to consume alcohol and drugs than those in civilian families [26], this could potentially suggest that children in military families are vulnerable to substance use problems at a certain age. Children with a sibling in the military were also significantly more likely to consume alcohol than children with a parent in the military [15]. This indicates that it may be beneficial for existing services for youth from military families, such as Families Overcoming Under Stress (FOCUS) and Military OneSource [43, 44], to offer advice and support for issues, such as child substance misuse, as an adjunct to the familial resilience intervention.

### Mental health

There was a lack of consistent evidence for the relationship between military connectedness and poorer mental health (i.e. suicidal ideation, depression, and PTSD) and low quality of life (i.e. perceived stress, positive affect, quality of life). Contributing to this was the notable lack of research regarding the relationship between military connectedness and mental health outcomes, for example only one study examined child PTSD [14]. Nonetheless, rates of mental health problems in military and non-military connected youth were generally consistent with nationally representative studies of US adolescents [13]. However, some evidence for elevated rates of mental health difficulties in youth with deployed parents compared to civilian parents was found (e.g. [14, 31]). This effect was more pronounced for males, with male children reporting significantly more depressive symptoms, poorer quality of life, and suicidal ideation compared to those with civilian parents [14]. This is notable as previous research has found such mental health problems to be generally more common in adolescent females than males in the general population (e.g. [45, 46]). Moreover, the deleterious impact of deployment on child psychological adjustment was not restricted to parental deployment and Cederbaum et al. [31] found an increased likelihood of depressive symptoms in youth who experienced the deployment of either a parent or a sibling compared to those who had not experienced familial deployment.

While most military connected children cope well, it is possible that the deployment of a family member can contribute towards the development of mental health difficulties in some children. This could be due to the stress experienced by young people when a family member deploys, such as the disruption of family routines, fears for the potential injury of the service member and uncertainty about the service member’s return [1, 47]. Familial reintegration following deployment can also be distressing for children due to the renegotiation of familial roles and psychological and/or physical injuries of the service member [48, 49].

### Strengths and limitations

This review was limited by several factors. First, no included study collected retrospective data on the child’s mental health. Second, most studies were cross-sectional (n = 8), school-based and reliant on child self-report. As a result, no data was collected regarding sample socio-economic status, deployment features (e.g. length of deployment, date of deployment) or family characteristics (e.g. family history of mental health problems). Therefore, it is unclear whether child wellbeing worsened immediately following parental deployment but later stabilised, if the elevated rates of child internalising and externalising problems are due to parental deployment or a comparatively challenging environment. Moreover, the collection of data regarding parental military status and wellbeing outcomes via child self-report may be subject to bias and future studies should include parent and/or teacher report. Third, several studies used data from the same large-scale public-school surveys and must be taken into consideration when interpreting the findings. Finally, all included studies were conducted in the US. Rates of mental health difficulties for civilian and military personnel children differ between the US and other nations, and the duration of deployment of US. AF is often longer than in other countries [13, 50, 51]. Therefore, the impact of parent or sibling military service on child wellbeing may be greater compared to non-US contexts, although this relationship requires further exploration.

Nonetheless, this review has several strengths, including the use of a thorough, systematic search strategy and the direct comparison of child wellbeing in military and non-military families. This review utilised a broad definition of what constitutes a “military family,” including a parent and/or a sibling in the AF. In doing so, we found having a sibling in the military to be significantly associated with higher rates of alcohol consumption compared to children with a military parent [15] as well as an increased likelihood of depressive symptoms in youth whose parent or sibling had deployed compared to those who had not experienced familial deployment [31]. This highlights the need for the impact of military family membership on child wellbeing to be examined more broadly, including not only parents but also siblings and other close relatives.

## Conclusions

We conducted a systematic review of the impact of familial military service on child wellbeing that is unique in its direct comparison of child outcomes in military and non-military families. On the whole, military connected youth were not found to have poorer wellbeing than children from civilian families, although those with deployed family members and older military connected children may be at somewhat greater risk of adjustment difficulties. Most research to date has focused on the parent–child relationship and the results of this study highlight the need for additional investigations of the impact of having a sibling and other close relatives in the military on child wellbeing. Given the cross-sectional nature of the included studies and the mixed evidence found, we suggest that other factors and influential moderator variables are considered in future research of child wellbeing.

### Key points

- This systematic review presents a unique direct comparison of child outcomes in military and non-military connected families to determine the impact of military family membership on child wellbeing.
- Few differences in wellbeing between children from military vs non-military families were observed. Children with deployed parents and older military connected children were at greater risk of some difficulties (e.g. substance use, violent, externalising behaviour).
- Having a sibling in the military and experiencing sibling deployment was statistically significantly associated with substance use and depressive symptoms.
- The results indicate that some children from military families may require additional support. Violence prevention programs and school-based support for military children may be beneficial in promoting child coping.

## Footnotes

1 Data from the 2008 HYS was used by Reed et al. [14, 26]. For the present study, data regarding child suicidal ideation and poor quality of life was utilised from Reed et al. [14] and data regarding child violent behaviour and substance use was included from Reed et al. [26] to avoid potential overlap. Gilreath et al. [15] reported CHKS data on substance use in 7th, 9th and 11th graders recruited during February–March 2011. Cederbaum et al. [31] reported CHKS data on suicidal ideation, depression and positive affect in 7th, 9th, and 11th grade graders recruited during 2011. Gilreath et al. [29] used CHKS data on substance use collected from 9th to 11th graders during 2012–2013. Sullivan et al. [27] reported CHKS data on substance use and violent behaviour in 7th, 9th, and 11th grade students collected during March–April 2013.

## References

[1] Park N (2011). Military children and families: strengths and challenges during peace and war. Am Psychol.

[2] Liu J (2004). Childhood externalizing behavior: theory and implications. J Child Adolesc Psychiatr Nurs..

[3] Keiley MK, Bates JE, Dodge KA, Pettit GS (2000). A cross-domain growth analysis: externalizing and internalizing behaviors during 8 years of childhood. J Abnorm Child Psychol.

[4] Beyer T, Furniss T (2007). Child psychiatric symptoms in primary school. Soc Psychiatry Psychiatr Epidemiol.

[5] Aranda MC, Middleton LS, Flake E, Davis BE (2011). Psychosocial screening in children with wartime-deployed parents. Mil Med.

[6] Kelley ML (1994). The effects of military-induced separation on family factors and child behavior. Am J Orthopsychiatry.

[7] Barker LH, Berry KD (2009). Developmental issues impacting military families with young children during single and multiple deployments. Mil Med.

[8] Fear NT, Reed RV, Rowe S, Burdett H, Pernet D, Mahar A, Iversen AC, Ramchandani P, Stein A, Wessely S (2018). Impact of paternal deployment to the conflicts in Iraq and Afghanistan and paternal post-traumatic stress disorder on the children of military fathers. Br J Psychiatry..

[9] Kelley ML, Hock E, Smith KM, Jarvis MS, Bonney JF, Gaffney MA (2001). Internalizing and externalizing behavior of children with enlisted navy mothers experiencing military-induced separation. J Am Acad Child Adolesc Psychiatry.

[10] Jensen PS, Martin D, Watanabe H (1996). Children’s response to parental separation during operation desert storm. J Am Acad Child Adolesc Psychiatry.

[11] Chassin L, Pitts SC (2002). Binge drinking trajectories from adolescence to emerging adulthood in a high-risk sample: predictors and substance abuse outcomes Adult and Family Development Project View project. J Consult Clin Psychol.

[12] Stone AL, Becker LG, Huber AM, Catalano RF (2012). Review of risk and protective factors of substance use and problem use in emerging adulthood. Addict Behav.

[13] Merikangas KR, He J-P, Burstein M, Swanson SA, Avenevoli S, Cui L (2010). Lifetime prevalence of mental disorders in US adolescents: results from the National Comorbidity Survey Replication-Adolescent Supplement (NCS-A). J Am Acad Child Adolesc Psychiatry..

[14] Reed SC, Bell JF, Edwards TC (2011). Adolescent well-being in Washington state military families. Am J Public Health.

[15] Gilreath TD, Cederbaum JA, Astor RA, Benbenishty R, Pineda D, Atuel H (2013). Substance use among military-connected youth. Am J Prev Med.

[16] Leen-Feldner EW, Feldner MT, Knapp A, Bunaciu L, Blumenthal H, Amstadter AB (2013). Offspring psychological and biological correlates of parental posttraumatic stress: review of the literature and research agenda. Clin Psychol Rev..

[17] Mayberry ML, Espelage DL, Koenig B (2009). Multilevel modeling of direct effects and interactions of peers, parents, school, and community influences on adolescent substance use. J Youth Adolesc.

[18] Feinberg ME, Solmeyer AR, McHale SM (2012). The third rail of family systems: sibling relationships, mental and behavioral health, and preventive intervention in childhood and adolescence. Clin Child Fam Psychol Rev.

[19] Rodriguez AJ, Margolin G (2011). Siblings of military servicemembers: a qualitative exploration of individual and family systems reactions. Prof Psychol Res Pract..

[20] Mitchell C, Brooks-Gunn J, Garfinkel I, McLanahar S, Notterman D, Hobcraft J (2015). Family structure instability, genetic sensitivity, and child well-being. AJS..

[21] Ronen T, Hamama L, Rosenbaum M, Mishely-Yarlap A (2016). Subjective well-being in adolescence: the role of self-control, social support, age, gender, and familial crisis. J Happiness Stud.

[22] Williamson, V, Cresswell, C, Fearon, P, Hiller, R, Walker, J, Halligan S. The role of parenting behaviors in childhood post-traumatic stress disorder: a meta-analysis and systematic review. Submitt Publ. 2016. 10.1016/j.cpr.2017.01.005 28137661

[23] Moher D, Liberati A, Tetzlaff J, Altman DG (2009). Preferred Reporting Items for Systematic Reviews and Meta-Analyses: the PRISMA statement (Reprinted from Annals of Internal Medicine). Phys Ther.

[24] Barnes VA, Davis H, Treiber FA (2007). Perceived stress, heart rate, and blood pressure among adolescents with family members deployed in Operation Iraqi Freedom. Mil Med.

[25] NIH (2014). Assessment tool for observational cohort and cross-sectional studies.

[26] Reed SC, Bell JF, Edwards TC (2014). Weapon carrying, physical fighting and gang membership among youth in Washington state military families. Matern Child Health J.

[27] Sullivan K, Capp G, Gilreath TD, Benbenishty R, Roziner I, Astor RA (2015). Substance abuse and other adverse outcomes for military-connected youth in California: results from a large-scale normative population survey. JAMA Pediatr..

[28] Acion L, Ramirez MR, Jorge RE, Arndt S (2013). Increased risk of alcohol and drug use among children from deployed military families. Addiction.

[29] Gilreath TD, Wrabel SL, Sullivan KS, Capp GP, Roziner I, Benbenishty R (2016). Suicidality among military-connected adolescents in California schools. Eur Child Adolesc Psychiatry.

[30] Reinhardt J, Clements-Nolle K, Yang W (2016). Physical fighting among male and female adolescents of military families. J Interpers Violence.

[31] Cederbaum JA, Gilreath TD, Benbenishty R, Astor RA, Pineda D, DePedro KT (2014). Well-being and suicidal ideation of secondary school students from military families. J Adolesc Health.

[32] US Department of Defence (2008). Contingency tracking system deployment file.

[33] CA Dept. of Ed. California Healthy Kids Survey, Student Well-being in California, 2008–10: Statewide Elementary Results. 2013.

[34] Weathers FW, Litz BT, Herman DS, Huska JA, Keane TM. The PTSD Checklist (PCL): reliability, validity, and diagnostic utility. In: Pap Present Annu Meet Int Soc Trauma Stress Stud San Antonio, TX. 1993.

[35] Norris FH (2002). Disasters in urban context. J Urban Health Bull New York Acad Med..

[36] Topolski TD, Patrick DL, Edwards TC, Huebner CE, Connell FA, Mount KK (2001). Quality of life and health-risk behaviors among adolescents. Journal Adolesc Health.

[37] Forbis SG, McAllister TR, Monk SM, Schlorman CA, Stolfi A, Pascoe JM (2007). Children and firearms in the home: a Southwestern Ohio Ambulatory Research Network (SOAR-Net) study. J Am Board Fam Med..

[38] Abuse TNC on A and S. No Title. 2011.

[39] Jang SJ, Ferguson TW, Rhodes JR (2016). Does alcohol or delinquency help adolescents feel better over time? A study on the influence of heavy drinking and violent/property offending on negative emotions. Int J Offender Ther Comp Criminol..

[40] Barnes GM, Hoffman JH, Welte JW, Farrell MP, Dintcheff BA (2006). Effects of parental monitoring and peer deviance on substance use and delinquency. J Marriage Fam..

[41] Westling E, Andrews JA, Hampson SE, Peterson M (2008). Pubertal timing and substance use: the effects of gender, parental monitoring and deviant peers. J Adolesc Health.

[42] Lara-Cinisomo S, Chandra A, Burns RM, Jaycox LH, Tanielian T, Ruder T (2012). A mixed-method approach to understanding the experiences of non-deployed military caregivers. Matern Child Health J.

[43] Lester P, Liang LJ, Milburn N, Mogil C, Woodward K, Nash W, Aralis H, Sinclair M, Semaan A, Klosinski L, Beardslee W (2016). Evaluation of a family-centered preventive intervention for military families: parent and child longitudinal outcomes. J Am Acad Child Adolesc Psychiatry.

[44] Esposito-Smythers C, Wolff J, Lemmon KM, Bodzy M, Swenson RR, Spirito A (2011). Military youth and the deployment cycle: emotional health consequences and recommendations for intervention. J Fam Psychol.

[45] Cash SJ, Bridge JA (2009). Epidemiology of youth suicide and suicidal behavior. Curr Opin Pediatr.

[46] Breslau J, Gilman SE, Stein BD, Ruder T, Gmelin T, Miller E (2017). Sex differences in recent first-onset depression in an epidemiological sample of adolescents. Transl Psychiatry..

[47] Lester P, Peterson K, Reeves J, Knauss L, Glover D, Mogil C (2010). The long war and parental combat deployment: effects on military children and at-home spouses. J Am Acad Child Adolesc Psychiatry.

[48] Bello-Utu CF, DeSocio JE (2015). Military deployment and reintegration: a systematic review of child coping. J Child Adolesc Psychiatr Nurs..

[49] Cozza SJ, Guimond JM, McKibben JBA, Chun RS, Arata-Maiers TL, Schneider B (2010). Combat-injured service members and their families: The relationship of child distress and spouse-perceived family distress and disruption. J Trauma Stress.

[50] Hunt EJF, Wessely S, Jones N, Rona RJ, Greenberg N (2014). The mental health of the UK Armed Forces: where facts meet fiction. Eur J Psychotraumatol..

[51] Sundin J, Herrell RK, Hoge CW, Fear NT, Adler AB, Greenberg N, et al. Mental health outcomes in US and UK military personnel returning from Iraq. Br J Psychiatry. 2014. http://bjp.rcpsych.org/content/early/2014/01/02/bjp.bp.113.129569.short. Accessed 10 May 2017. 10.1192/bjp.bp.113.129569 24434071

